# 

**Published:** 1906-12

**Authors:** 


Sixty-second Year.

Vol. LXII. DECEMBER, 1906. No. 5

### BUFFALO MEDICAL JOURNAL

Established 1845 by AUSTIN FLINT, M. D.

EDITOR:

WILLIAM WARREN POTTER, M. D.

ASSISTANT EDITORS:

WILLIAM C. KRAUSS, M. D. NELSON W. WILSON, M. D.

ASSOCIATE EDITORS:

JAMES WRIGHT PUTNAM, M. D. ERNEST WENDE, M. D.
JOHN PARMENTER, M. D. JOHN A. MILLER, Ph. D.
HARVEY R. GAYLORD, M. D. MAUD J. FRYE, M. D.
JOHN A. RAFTER, M. D.

